# Identification of Bacterial Communities and Tick-Borne Pathogens in *Haemaphysalis* spp. Collected from Shanghai, China

**DOI:** 10.3390/tropicalmed7120413

**Published:** 2022-12-01

**Authors:** Wenbo Zeng, Zhongqiu Li, Tiange Jiang, Donghui Cheng, Limin Yang, Tian Hang, Lei Duan, Dan Zhu, Yuan Fang, Yi Zhang

**Affiliations:** 1 National Institute of Parasitic Diseases, Chinese Center for Disease Control and Prevention (Chinese Center for Tropical Diseases Research), NHC Key Laboratory of Parasite and Vector Biology, WHO Collaborating Center for Tropical Diseases, National Center for International Research on Tropical Diseases, Shanghai 200025, China; 2 School of Global Health, Chinese Center for Tropical Diseases Research, Shanghai Jiao Tong University School of Medicine, Shanghai 200025, China

**Keywords:** Ixodidae, bacteria, TBPs, metagenomics, PCR, China

## Abstract

Ticks can carry and transmit a large number of pathogens, including bacteria, viruses and protozoa, posing a huge threat to human health and animal husbandry. Previous investigations have shown that the dominant species of ticks in Shanghai are *Haemaphysalis flava* and *Haemaphysalis longicornis*. However, no relevant investigations and research have been carried out in recent decades. Therefore, we investigated the bacterial communities and tick-borne pathogens (TBPs) in *Haemaphysalis* spp. from Shanghai, China. Ixodid ticks were collected from 18 sites in Shanghai, China, and identified using morphological and molecular methods. The V3–V4 hypervariable regions of the bacterial 16S rRNA gene were amplified from the pooled tick DNA samples and subject to metagenomic analysis. The microbial diversity in the tick samples was estimated using the alpha diversity that includes the observed species index and Shannon index. The Unifrac distance matrix as determined using the QIIME software was used for unweighted Unifrac Principal coordinates analysis (PCoA). Individual tick DNA samples were screened with genus-specific or group-specific nested polymerase chain reaction (PCR) for these TBPs and combined with a sequencing assay to confirm the results of the V3–V4 hypervariable regions of the bacterial 16S rRNA gene. We found *H. flava* and *H. longicornis* to be the dominant species of ticks in Shanghai in this study. Proteobacteria, Firmicutes, Bacteroidetes and Actinobacteria are the main bacterial communities of *Haemaphysalis* spp. The total species abundances of Proteobacteria, Firmicutes and Bacteroidetes, are 48.8%, 20.8% and 18.1%, respectively. At the level of genus analysis, *H. longicornis* and *H. flava* carried at least 946 genera of bacteria. The bacteria with high abundance include *Lactobacillus*, *Coxiella*, *Rickettsia* and *Muribaculaceae*. Additionally, *Rickettsia rickettsii*, *Rickettsia japonica*, *Candidatus Rickettsia jingxinensis*, *Anaplasma bovis*, *Ehrlichia ewingii*, *Ehrlichia chaffeensis*, *Coxiella* spp. and *Coxiella*-like endosymbiont were detected in *Haemaphysalis* spp. from Shanghai, China. This study is the first report of bacterial communities and the prevalence of some main pathogens in *Haemaphysalis* spp. from Shanghai, China, and may provide insights and evidence for bacterial communities and the prevalence of the main pathogen in ticks. This study also indicates that people and other animals in Shanghai, China, are exposed to several TBPs.

## 1. Introduction

Ticks are the second largest infectious agent in the world after mosquitoes, with a wide variety of species and a wide range of animal hosts [1]. They can carry and transmit a large number of pathogens, including bacteria, viruses and protozoa, posing a huge threat to human health and animal husbandry [2]. Ticks are known to harbor a number of veterinary and medically important bacterial species within the *Rickettsia*, *Anaplasma*, *Bartonella*, *Coxiella* and *Ehrlichia* genera [2,3]. As most ticks exhibit two-host or three-host life cycles, they are capable of supporting the transmission of pathogens between hosts, in which humans frequently serve as accidental hosts [4]. Multiple pathogenic agents may also be carried by an individual tick, which could transmit these pathogens to the human hosts bitten by ticks [3,4]. The main tick-borne diseases reported in China are forest encephalitis, Crimean Congo hemorrhagic fever, Lyme disease, rabbit fever, Q fever, North Asian tick-borne spotted fever, rickettsiosis, ehrlichiosis, anaplasmosis, babesiosis and Taylor disease [5,6,7,8]. Ticks of *Haemaphysalis* spp. have been implicated as potential disease vectors to humans and animals worldwide [3,9]. Various pathogenic bacteria have been previously detected in *Haemaphysalis* spp., including the disease agents for rickettsia spotted fever, tick typhus, anaplasmosis and ehrlichiosis [3,10,11,12].

Traditional detection methods (PCR, culture and serological methods), which have played a very important role in previous pathogen detection and identification, cannot detect all pathogens, especially when in low abundance and with unknown pathogens. Additionally, traditional detection methods are extremely dependent on known pathogens. However, metagenomic sequencing can identify a large number of micro pathogens, including unknown pathogens, in tick microflora captured in the field and does not depend on known nucleic acid sequences [13,14,15]. This method can be used not only to monitor microbial communities in infectious insect vectors but also as an ideal tool for monitoring emerging tick-borne diseases [13,15]. It can also provide a more thorough understanding of the ecological factors related to the prevalence and persistence of the vector-related microbial pedigree, which will help to predict and prevent the spread of diseases [13,15,16,17].

More and more tick-borne diseases and pathogens have been discovered. Recently, a new species of Yezo virus of the genus Nairobi virus was discovered in ticks in Hokkaido, Japan, and caused multiple infections [18]. Since the early 1980s, 34 pathogens of tick-borne diseases have been identified in mainland China, including eight species of spotted fever group *Rickettsia*, seven species of *Anaplasma*, six species of *Borrelia burgdorferi*, 11 species of *Babesia* and severe fever with thrombocytopenia syndrome new Bunyavirus (SFTSV) and Alongshan Virus (ALSV) [7,19]. Tick-borne diseases and tick bites frequently occur in provinces and cities around Shanghai, China, which seriously endangers the life and health of local residents. Human granulocytic anaplasmosis was first reported in Anhui Province in 2006. From 2010 to 2015, 286 cases of severe fever with thrombocytopenia syndrome (SFTS) were diagnosed in Jiangsu and Anhui provinces, with a fatality rate of 16.1% [20]. Li et al. (2021) predicted that most areas of Shanghai are highly suitable for ticks, and previous investigations have shown that the dominant ticks in Shanghai are *H. flava* and *H. longicornis* [21]. However, no relevant investigations and research have been undertaken in recent decades. Therefore, we expect that the metagenomic analysis of *Haemaphysalis* spp. in this region may provide an extensive list of pathogens carried by this important vector, thereby highlighting the potential risk of human infection caused by tick bites.

In our study, we first investigate the bacterial variability between populations of *H. flava* and *H. longicornis* from Shanghai, China. We sequenced the amplicons of the eubacterial 16S rRNA to (1) determine the baseline bacterial diversity from ticks collected from within a relatively small geographic area, (2) confirm the species identity of key taxa using taxon-specific PCR and Sanger sequencing and (3) estimate the relative abundance of the key bacterial taxa by PCR in the pooled DNA of ticks collected from Shanghai, China.

## 2. Materials and Methods

### 2.1. Study Area

**Figure 1:**
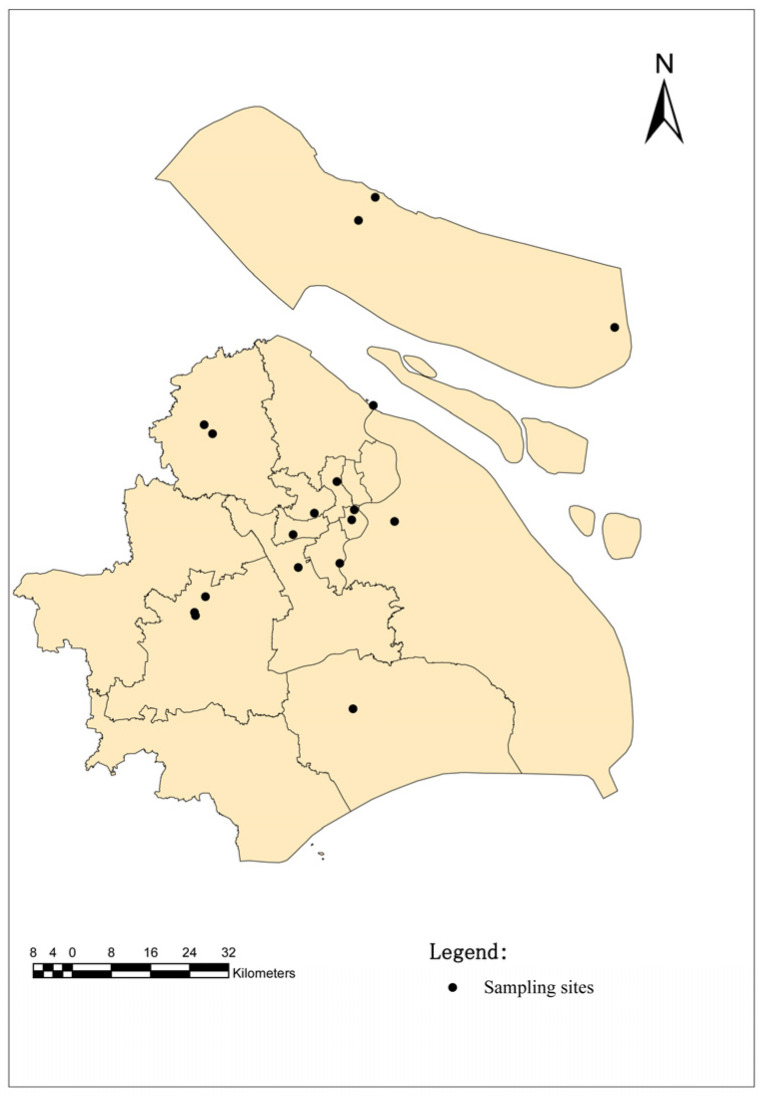
Map of the sampling sites in Shanghai, China. The black dots indicate the sampling regions in this study.

These ticks were collected from 18 sampling sites in Shanghai. Shanghai, China, is located in the front of the alluvial plain of the Yangtze River Delta, with soft soil and low and flat topography, with an average elevation of 4 m from east to west. Except for a few hills nearly 100 m above sea level in the west, Shanghai is a large and low-level plain, and the whole plain river port is similar to a net. Shanghai, China, belongs to the northern subtropical humid monsoon climate zone, warm, humid and rainy, with four distinct seasons. A suitable natural environment provides favorable conditions for the survival of ticks. The sample points are selected from random sites in each functional area of the city (Figure 1). (Central cities (CC): 121°45′ E, 31°16′ N; 121°37′ E, 31°19′ N; 121°47′ E, 31°22′ N; 121°45′ E, 31°28′ N; 121°41′ E, 31°23′ N; 121°48′ E, 31°24′ N; Out suburban districts (OSD): 121°21′ E, 31°11′ N; 121°19′ E, 31°08′ N; 121°48′ E, 30°92′ N; 121°18′ E, 31°07′ N; Inner suburban districts (ISD): 121°33′ E, 31°14′ N; 121°21′ E, 31°37′ N; 121°52′ E, 31°41′ N; 121°38′ E, 31°15′ N and Chongming island (CMI): 121°49′ E, 31°71′ N; 121°51′ E, 31°72′ N; 121°47′ E, 31°69′ N; 121°52′ E, 31°73′ N).

### 2.2. Ticks Collection and Species Identification

Wild ticks were collected by dragging flags on the vegetation layer during the day. Additionally, parasitic ticks (90 *H. flava* and 22 *H. longicornis*) were collected from animals (dogs, goats, sheep, cows, etc.). For the first step, different morphological characteristics were observed to identify the species and development stages of the collected ticks by an entomologist (Zhu Dan) [22], and then 12S rDNA [23] and Cytochrome C oxidase subunit I (CO I) gene [24] identification was used to further determine the species of ticks, as previously described. All ticks of the genus *Haemaphysalis* from each site were included in the study. Secondly, in the lab stage, the ticks were rinsed with 75% ethanol for 1 min to remove any environmental contaminants; then, they were rinsed with deionized water for 5 min to remove 75% ethanol and finally stored in a refrigerator (−80 °C).

### 2.3. DNA Extraction

After morphological identification, the ticks collected from the same site were pooled according to developmental stages. Overall, 2102 *H. flava* (80 adults, 295 nymphs and 1727 larvae) were divided into 211 pools, and 151 *H. longicornis* (65 adults, 8 nymphs and 78 larvae) were divided into 15 pools. The total genomic DNA was extracted using the DNeasy Blood & Tissue Kit (Qiagen, Hilden, Germany) following the manufacturer’s instructions. The DNA concentration and integrity were measured using a NanoDrop 2000 spectrophotometer (Thermo Fisher Scientific, Waltham, MA, USA) and agarose gel electrophoresis, respectively.

### 2.4. Molecular Identification of Tick Vectors by PCR

To further confirm the results of the morphological classification of *Haemaphysalis* spp., multi-locus sequence typing depending on five tick’s genomic DNA markers amplified fragments was carried out, which included: one nuclear gene CO I gene and a mitochondrial gene 12S rRNA. The PCR primers for the two genes are presented in Table 1.

**Table 1:** Primers used in this study.

Target	Gene	Primer Name	Sequence (5′-3′)	Reference
Tick	12S rDNA	T1B	AAACTAGGATTAGATACCCT	[23]
		T2A	AATGAGAGCGACGGGCGATGT	
Tick	CO I	HCO2198	TAAACTTCAGGGTGACCAAAAAATCA	[24]
		LCO1490	GGTCAACAAATCATAAAGATATTGG	
Microbiome	16S rDNA	343F	TACGGRAGGCAGCAG	[25]
		798R	AGGGTATCTAATCCT	
*Coxiella* spp.	16S rDNA	Cox16SF1	CGTAGGAATCTACCTTRTAGWGG	[16]
		Cox16SR1	ACTYYCCAACAGCTAGTTCTCA	
		Cox16SF2	TGAGAACTAGCTGTTGGRRAGT	
		Coc16SR2	GCCTACCCGCTTCTGGTACAATT	
*Rickettsia* spp.	ompA	Rr190.70p	ATGGCGAATATTTCTCCAAAA	[26]
		Rr190.602n	AGTGCAGCATTCGCTCCCCCT	
*Ehrlichia* spp.	16S rRNA	Eh-out1	TTGAGAGTTTGATCCTGGCTCAGAACG	[16,27]
		Eh-out2	CACCTCTACACTAGGAATTCCGCTATC	
		Eh-gs1	GTAATACTGTATAATCCCTG	
		Eh-gs2	GTACCGTCATTATCTTCCCTA	
*Anaplasma* spp.	16S rRNA	Eh-out1	TTGAGAGTTTGATCCTGGCTCAGAACG	[27]
		Eh-out2	CACCTCTACACTAGGAATTCCGCTATC	
		HGA1	GTCGAACGGATTATTCTTTATAGCTTG	
		HGA2	TATAGGTACCGTCATTATCTTCCCTAC	

### 2.5. DNA Amplification

PCR amplification of the V3–V4 hypervariable regions of the bacterial 16S rRNA gene was carried out in a 25 μL reaction system using universal primer pairs (343F and 789R) (Table 1). The reverse primer contained a sample barcode, and both primers were connected with an Illumina sequencing adapter (Illumina Inc., San Diego, CA, USA).

### 2.6. Library Construction and Sequencing

The Amplicon quality was visualized using gel electrophoresis. The PCR products were purified with Agencourt AMPure XP beads (Beckman Coulter Co., Breya, CA, USA) and quantified using a Qubit dsDNA assay kit. The concentrations were then adjusted for sequencing. The sequencing was performed on an Illumina NovaSeq6000 with two paired-end read cycles of 250 bases each (Illumina Inc.; OE Biotech Company, Shanghai, China).

### 2.7. Bioinformatics Analysis

Raw sequencing data were in the FASTQ format. Paired-end reads were then pre-processed using cutadapt software to detect and cut off the adapter. After trimming, the paired-end reads were filtered for low-quality sequences, denoised, merged and detected and cut off the chimera reads using DADA2 [28] with the default parameters of QIIME2 [29] (November 2020); last, the software outputs, the representative reads and the ASV abundance table was generated. The representative read of each ASV was selected using the QIIME 2 package. All of the representative reads were annotated and blasted against Silva database Version 138 using q2-feature-classifier with the default parameters. The microbial diversity in the tick samples was estimated using the alpha diversity that includes the observed species index and Shannon index. The Unifrac distance matrix performed by QIIME software was used for unweighted Unifrac Principal coordinates analysis (PCoA).

### 2.8. Specific PCR for Detection of Some Pathogens in Ticks

Based on the results of 16S rRNA gene amplicon sequencing, genus-/group-specific PCR was performed to confirm the presence of TBPs in individual ticks. PCR was performed using a PCR System 9700 (Applied Biosystems, GeneAmp^®^, Carlsbad, CA, USA). For PCR, 2 μL of each DNA sample (150–330 ng) was used as the template for the first round, and 1 μL of the primary PCR product was used as the template for the second round. For the first round, a negative control (ddwater) and an extraction control mentioned above were included in each PCR experiment. Tube strips with individual caps were used in the amplification steps to prevent cross-contamination, and all PCR amplifications were carried out using PrimeSTAR^®^ HS (Premix) (TaKaRa, Beijing, China). All of the operations were carried out in a biological safety cabinet. The amplified products were then electrophoresed on a 1.5% agarose gel, and the positive amplicons were sent to TSINGKE Biological Technology (Beijing, China) for sequencing. The PCR primers for *Rickettsia* spp., *Anaplasma* spp. and *Ehrlichia* spp. and *Coxiella* spp. are presented in Table 1.

### 2.9. Phylogenetic Analysis

The obtained nucleotide sequences were compared with those available in GenBank using the National Center for Biotechnology Information (NCBI; Bethesda, MD, USA) Basic Local Alignment Search Tool (BLAST) search engine (http://blast.ncbi.nlm.nih.gov/blast.cgi, accessed on 30 October 2022), and multiple sequence alignment was performed using the MEGA X (version 10.0) multiple alignment tool with the default parameters in MEGA X. The phylogenetic analysis was performed using MEGA X, and the tree was constructed using neighbor-joining (NJ) methods. The phylogenetic analysis of 12S rRNA and CO I gene for ticks, *ompA* for *Rickettsia* spp., 16S rRNA for *Anaplasma* spp., 16S rRNA for *Ehrlichia* spp., and 16S rRNA for *Coxiella* spp. was performed using the neighbor-joining method (NJ method) based on MEGA X. Bootstrap values were estimated for 1000 replicates.

## 3. Results

### 3.1. Taxonomic Classification and Sequencing Data Statistics

A total of 2253 hard ticks were identified as *H. flava* (*n* = 2102) and *H. longicornis* (*n* = 151) based on morphological identifications and confirmed by species-specific PCR and sequencing assays. There were 20 groups of *H. flava* (CCF1-5, OSDF1-5, ISDF1-5 and CMIF1-5) and 10 groups of *H. longicornis* (CCL1-5 and CMIL1-5) analyzed by amplicon sequencing on an Illumina NovaSeq6000 platform. The raw read data of the sequencing machine were distributed between 78,084 and 81,941, and the clean tag data after quality control were distributed between 4449 and 67,697. The valid tags (the final data used for analysis) data of clean tags were distributed between 4395 and 64,359, and the Amplicon Sequence Variant (ASV) numbers of each sample were distributed between 16 and 1191.

### 3.2. Alpha Diversity

**Figure 2:**
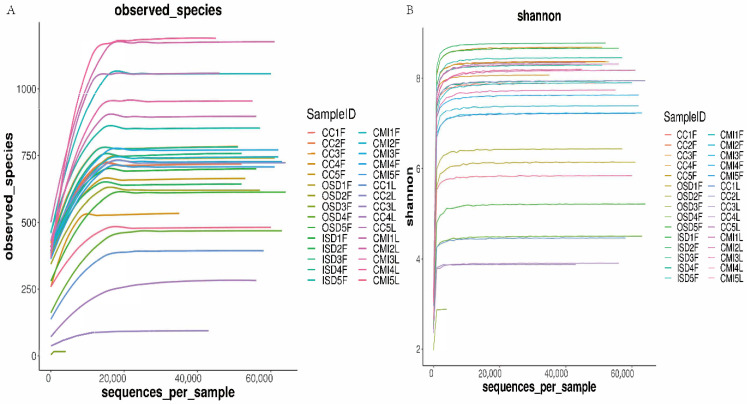
Rarefaction curves of observed species (**A**) and Shannon diversity index (**B**) for group tick samples.

Rarefaction curves were obtained for each tick group to determine if the sequencing depth was sufficient for each sample. Although the rarefaction curves for the observed ASVs approached saturation (Figure 2A), the Shannon diversity index curves reached a stable value (Figure 2B). The Good’s coverage values for each group of samples ranged from 91.69% to 100%, indicating that the majority of the ASVs had been discovered. Altogether, these results suggest that the sequencing depth was sufficient to represent the majority of bacterial communities in these samples.

### 3.3. Bacterial Microbiome Composition

All of the valid sequences were classified with 100% identity, and species information was obtained by comparison with the SILVA-138SSUrRNA database. A total of 15,903 ASVs were detected in this study, with a total of 1872 species belonging to 33 phyla, 91 classes, 235 orders, 403 families and 946 genera. A total of 10674ASVs were detected in *H. flava*, with a total of 1872 species belonging to 32 phyla, 84 classes, 209 orders, 358 families and 801 genera. A total of 6010ASVs were detected in *H. longicornis*, with a total of 1162 species belonging to 31 phyla, 81 classes, 201 orders, 333 families and 696 genera.

Proteobacteria, Firmicutes, Bacteroidetes and Actinobacteria are the main components of bacterial communities of *Haemaphysalis* spp. The total species abundances of Proteobacteria, Firmicutes and Bacteroidetes, are 48.80%, 20.80% and 18.10%, respectively.

**Figure 3:**
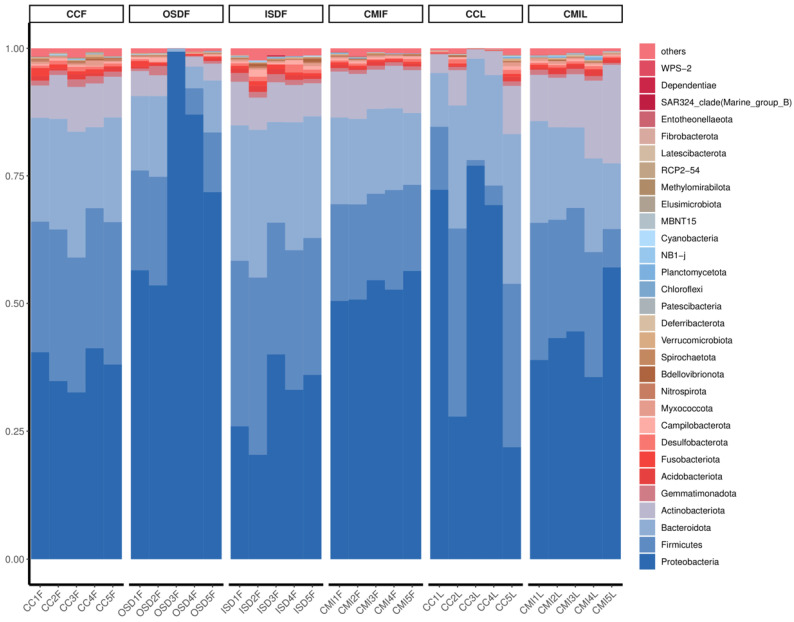
Relative abundances of potential top 30 pathogens at the phylum level in grouped *H. flava* and *H. longicornis* samples. The groups of *H. flava* are CCF, OSDF, ISDF and CMIF, and the groups of *H. longicornis* are CCL and CMIL.

The bacteria in *H. flava* with high abundance include Proteobacteria, 48.80%; Firmicutes, 21.60%; Bacteroidetes, 17.70% and Actinobacteria, 6.70%. The bacteria in *H. longicornis* with high abundance include Proteobacteria, 48.8%; Firmicutes, 19.20%; Bacteroidetes, 19.00% and Actinobacteria, 9.00% (Figure 3).

**Figure 4:**
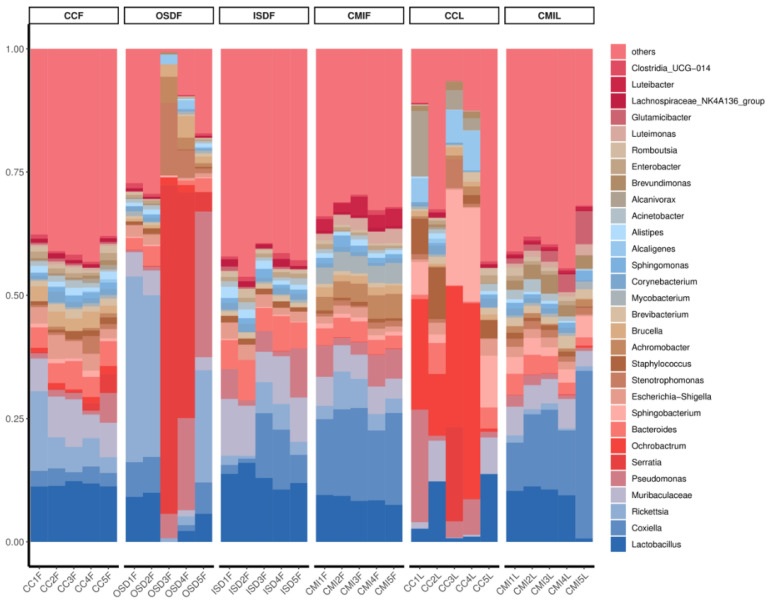
Relative abundances of potential top 30 pathogens at genus level in grouped *H. flava* and *H. longicornis* samples. The groups of *H. flava* are CCF, OSDF, ISDF and CMIF and the groups of *H. longicornis* are CCL and CMIL.

At the level of genus analysis, *H. longicornis* and *H. flava* carried at least 946 genera of bacteria. The bacteria in *H. flava* with high abundance include *Lactobacillus*, *Rickettsia*, *Coxiella*, *Serratia* and *Muribaculaceae*. The bacteria in *H. longicornis* with high abundance include *Ochrobactrum*, *Coxiella*, *Lactobacillus* and *Sphingobacterium*. Additionally, some groups of OSDF, CCF and CCL include *Serratia* (Figure 4).

### 3.4. Species and Location-Specific Differences in Microbial Diversity

**Figure 5:**
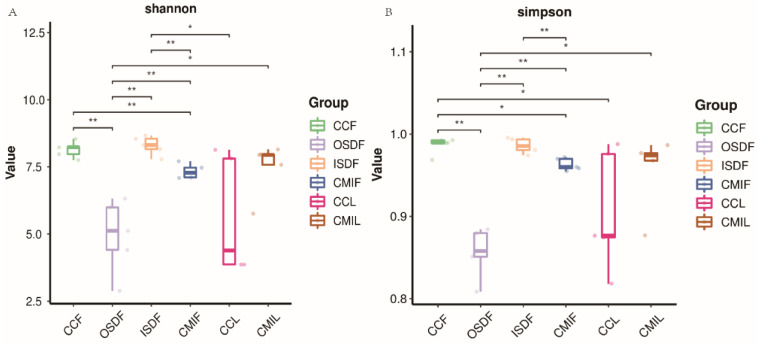
Plots of Alpha diversity indices of grouped *H. flava* and *H. longicornis* samples. Plot (**A**) the Shannon diversity index and plots (**B**) the Simpson diversity index (* *p* < 0.05, ** *p* < 0.01). The groups of *H. flava* are CCF, OSDF, ISDF and CMIF and the groups of *H. longicornis* are CCL and CMIL.

**Figure 6:**
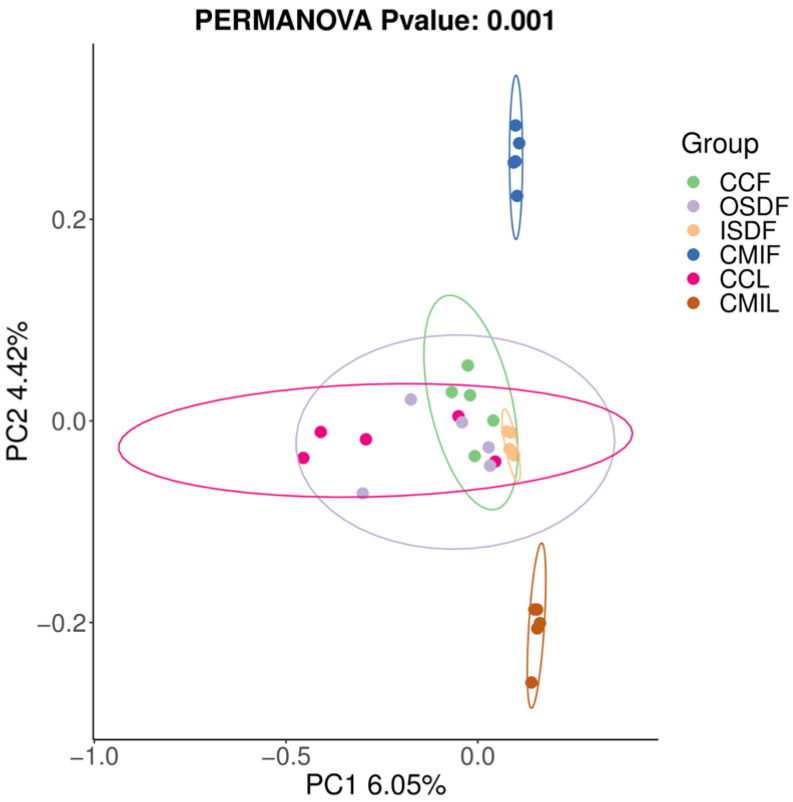
Principal coordinates analysis (PCoA) plot of 16SrRNA data from grouped samples of *H. flava* and *H. longicornis* samples of sample-based ecological distance. The groups of *H. flava* are CCF, OSDF, ISDF and CMIF and the groups of *H. longicornis* are CCL and CMIL.

We examined the effects of location and tick species on the bacterial diversity of the grouped samples. Diversity indices CCF, ISDF, CMIF and CMIL clustered tightly, while OSDF and CCL were more diffused (Variable), and some of them were significant (Figure 5). In the principal coordinates analysis, decreased variability could be accounted for across two axes (6.05% × 4.42%) (Figure 6). We detected a significant difference in the bacterial community between locations and species.

### 3.5. Bacterial Relative Abundance Differences

**Figure 7:**
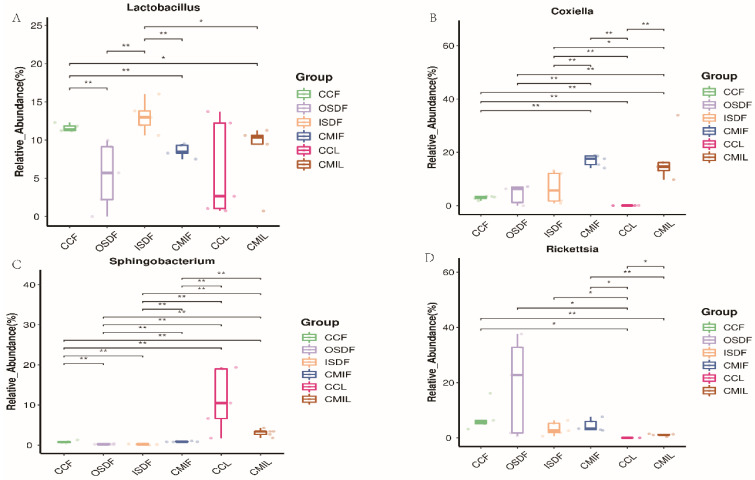
Relative titers of bacterial 16SrRNA gene from *Lactobacillus* (**A**), *Coxiella* (**B**), *Sphingobacterium* (**C**) and *Rickettsia* (**D**) between groups of *H. flava* and *H. longicornis* samples (* *p* < 0.05, ** *p* < 0.01). The groups of *H. flava* are CCF, OSDF, ISDF and CMIF and the groups of *H. longicornis* are CCL and CMIL.

Of special interest is the identification of members of the genera *Coxiella*, *Legionella*, *Anaplasma*, *Ehrlichia*, *Rickettsia* and *Sphingomonas*, some of which are pathogenic and can be transmitted by ticks. The relative abundances of *Lactobacillus*, *Coxiella*, *Sphingobacterium* and *Rickettsia* differed significantly between the two groups (Figure 7).

### 3.6. Prevalence of Tick-Borne Pathogens in Individual Pools

The important pathogenic bacterial genera *Rickettsia* and *Coxiella* were found in the grouped tick samples. Each pool was detected by the genus-/species-specific PCR combined with sequencing in order to identify the TBPs carried by it. In addition, *Anaplasma* and *Ehrlichia* were often detected in ticks, so each pool was screened by *Anaplasma*/*Ehrlichia*-specific PCR.

As a result, *R. rickettsii* (2.37%, 5/211), *R. japonica* (3.32%, 7/211), *Candidatus R. jingxinensis* (16.59%, 35/211), *A. bovis* (1.42%, 3/211), *E. ewingii* (0.95%, 2/211), *E. chaffeensis* (1.90%, 4/211), *Coxiella* spp. (1.90%, 4/211), *C.*-like endosymbiont (2.37%, 5/211) were detected in *H. flava.* from Shanghai and *R. rickettsii* (20.00%, 3/15), *R. japonica* (20.00%, 3/15), *Candidatus R. jingxinensis* (20.00%, 3/15), *A. bovis* (26.67%, 4/15), *E. ewingii* (13.33%, 2/15), *E. chaffeensis* (6.67%, 1/15), *Coxiella* spp. (6.67%, 1/15), *C.*-like endosymbiont (20.00%, 3/15) were also detected in *H. longicornis* from Shanghai (Table 2).

### 3.7. Symbiotic Interaction of Bacterial Communities

**Figure 8:**
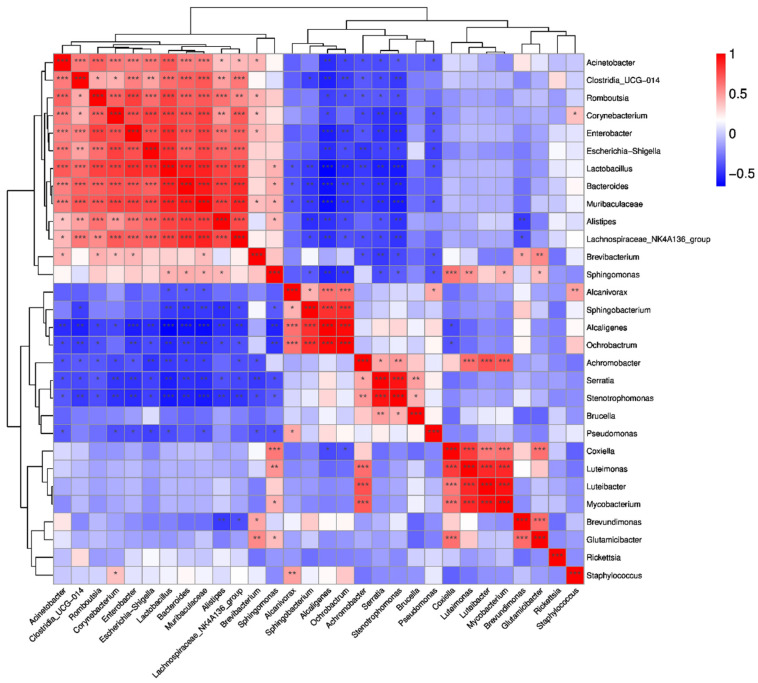
Correlation heatmap of Spearman coefficient of the top 30 genera of the groups of *H. flava* and *H. longicornis* samples (* *p* < 0.05, ** *p* < 0.01,*** *p* < 0.001). The groups of *H. flava* are CCF, OSDF, ISDF and CMIF and the groups of *H. longicornis* are CCL and CMIL.

The study also predicted the correlation among the top 30 genera in abundance. There is a positive correlation between *Acinetobacter*, *Clostridia_UGG-014*, *Romboutsia*, *Corynebacterium*, *Enterobacter*, *Escherichia-Shigella*, *Lactobacillus*, *Bacteroides*, *Muribaculaceae*, *Alistipes* and *Lachnospiraceae_NK4A136_group*. There is a negative correlation between one of *Alcaligenes*, *Orchrobactrum*, *Achromobacter*, *Serratia*, *Stenotrophomonas* and one of the above that has a positive correlation between them. There also is a positive correlation between *Achromobacter*, *Serratia*, *Stenotrophomonas* and *Brucella*, and there is a positive correlation between *Coxilla*, *Luteimonas*, *Luteibacter* and *Mycobacterium* (Figure 8).

### 3.8. Phylogenetic Analysis

**Figure 9:**
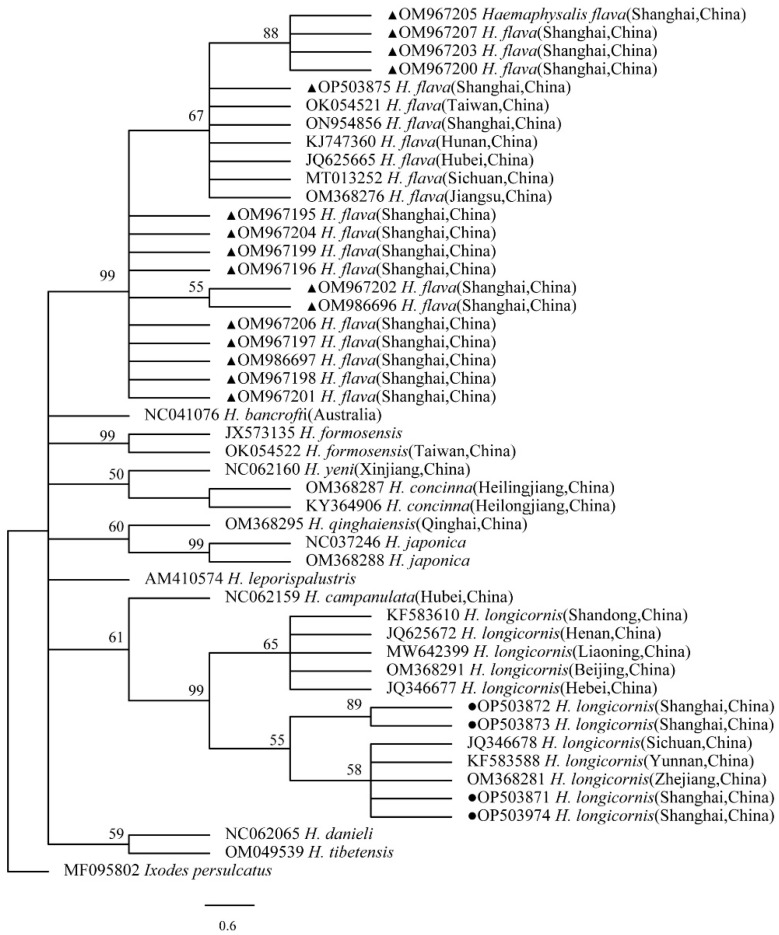
Phylogenetic tree of *H. flava* and *H. longicornis* based on partial 12S rDNA gene sequence similarity. The sequence from *H. flava* obtained in this study is indicated with a black triangle, and the sequence from *H. longicornis* obtained in this study is indicated with black dots. Sequences were aligned using the MEGA X (version 10.0) software package. Phylogenetic analysis was performed by the neighbor-joining method (NJ method), and bootstrap values were estimated for 1000 replicates. Kimura’s two-parameter model was used as a substitution model for the calculation of the phylogenetic trees.

**Figure 10:**
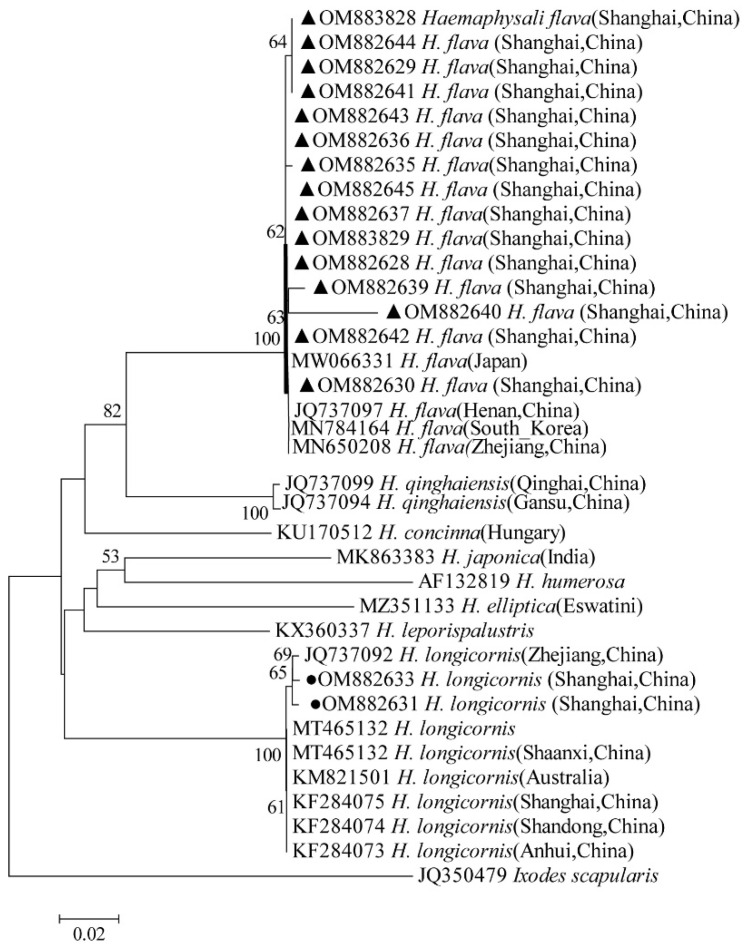
Phylogenetic tree of *H. flava* and *H. longicornis* based on partial CO I gene sequence similarity. The sequence from *H. flava* obtained in this study is indicated with a black triangle, and the sequence from *H. longicornis* obtained in this study is indicated with black dots. Sequences were aligned using the MEGA X (version 10.0) software package. Phylogenetic analysis was performed by the neighbor-joining method (NJ method), and bootstrap values were estimated for 1000 replicates. Kimura’s two-parameter model was used as a substitution model for the calculation of the phylogenetic trees.

**Figure 11:**
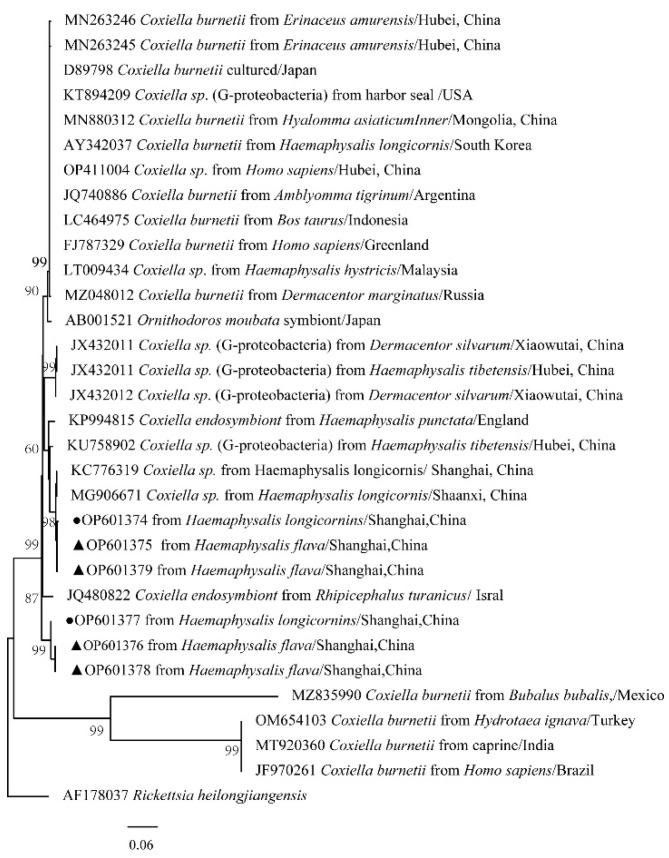
Phylogenetic tree of *Coxiella* spp. in ticks based on partial 16S rDNA gene sequence similarity. The sequence from *H. flava* obtained in this study is indicated with a black triangle, and the sequence from *H. longicornis* obtained in this study is indicated with black dots. Sequences were aligned using the MEGA X (version 10.0) software package. Phylogenetic analysis was performed by the neighbor-joining method (NJ method), and bootstrap values were estimated for 1000 replicates. The Kimura two-parameter model was used as a substitution model for the calculation of the phylogenetic trees.

**Figure 12:**
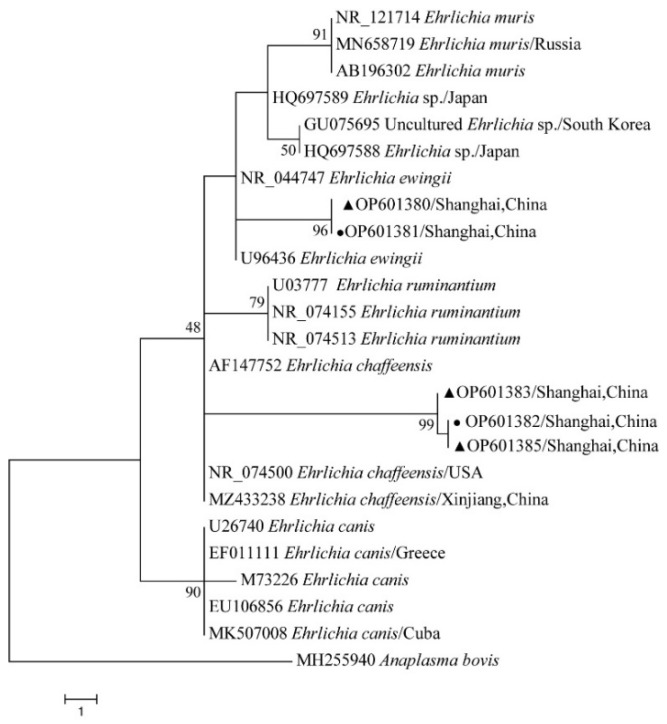
Phylogenetic tree of *Ehrlichia* spp. in ticks based on partial 16S rDNA gene sequence similarity. The sequence from *H. flava* obtained in this study is indicated with a black triangle, and the sequence from *H. longicornis* obtained in this study is indicated with black dots. Sequences were aligned by using the MEGA X (version 10.0) software package. Phylogenetic analysis was performed by the neighbor-joining method (NJ method), and bootstrap values were estimated for 1000 replicates. The Kimura two-parameter model was used as a substitution model for the calculation of the phylogenetic trees.

**Figure 13:**
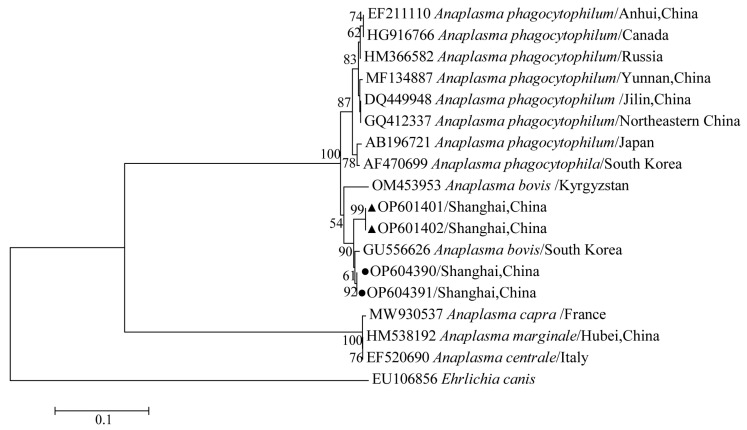
Phylogenetic tree of *Anaplasma* spp. in ticks based on partial 16S rDNA gene sequence similarity. The sequence from *H. flava* obtained in this study is indicated with a black triangle, and the sequence from *H. longicornis* obtained in this study is indicated with black dots. Sequences were aligned using the MEGA X (version 10.0) software package. Phylogenetic analysis was performed by the neighbor-joining method (NJ method), and bootstrap values were estimated for 1000 replicates. The Kimura two-parameter model was used as a substitution model for the calculation of the phylogenetic trees.

**Figure 14:**
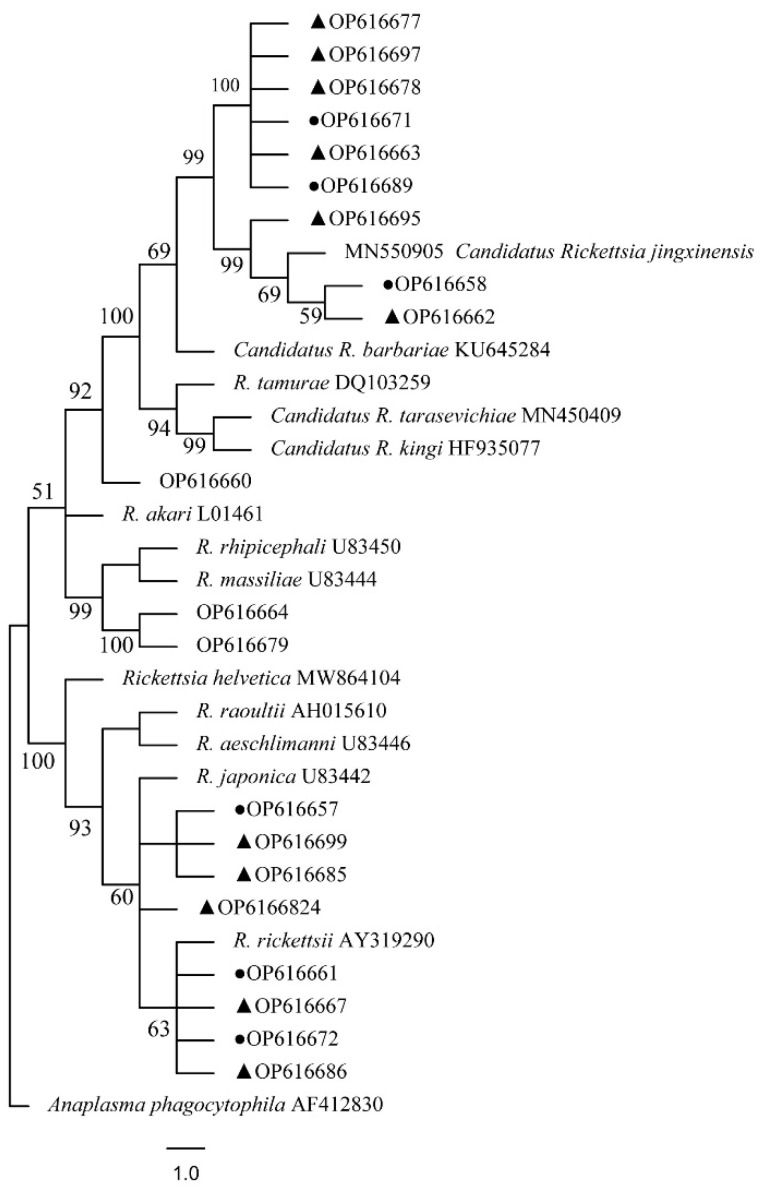
Phylogenetic tree of *Rickettsia spp.* in ticks based on partial *ompA* gene sequence similarity. The sequence from *H. flava* obtained in this study is indicated with a black triangle, and the sequence from *H. longicornis* obtained in this study is indicated with black dots. Sequences were aligned using the MEGA X (version 10.0) software package. Phylogenetic analysis was performed by the neighbor-joining method (NJ method), and bootstrap values were estimated for 1000 replicates. The Kimura two-parameter model was used as a substitution model for the calculation of the phylogenetic trees.

The phylogenetical analyses of 12S rDNA and CO I of the two tick species sequences (*H. flava* and *H. longicornis*) agreed with their morphological identification. Constructing a phylogenetic tree based on 12S rDNA, *H. flava* and *H. longicornis* were placed in the same clades with *H. flava* (OK054521, ON954856, KJ747360, JQ625665, MT013252, OM368276) and *H. longicornis* (JQ346678, KF583588, OM368281), respectively (Figure 9), and *H. flava* and *H. longicornis* were also placed in a clade with *H. flava* (MN066331, JQ737097, MN784164, MN650208) and *H. longicornis* (JQ737092), respectively, in the COI tree (Figure 10). By phylogenetic analysis, *Coxiella* spp. and *C.*-like endosymbiont, identified in both *H. flava* and *H. longicornis*, were shown to be clustered with *Coxiella* spp. (KC776319, MG906671) and *C.*-like endosymbiont (JQ480822), respectively (Figure 11). *E. ewingii* and *E. chaffeensis*, identified in both tick species in the study, were also placed in a clade with *E. ewingii* (NR_044747, U96436) and *E. chaffeensis* (AF147752, NR_074500, MZ433238) (Figure 12). *A. bovis*, identified in *H. flava* and *H. longicornis*, were shown to be clustered with *A. bovis* (GU556626) (Figure 13). *R. japonica*, *R. rickettsii* and *Candidatus R. jingxinensis*, identified in both tick species in the study, were also placed in a clade with *R. japonica* (U83440), *R. rickettsii* (AY319290) and *Candidatus R. jingxinensis* (MN550905) (Figure 14).

## 4. Discussion

In recent years, more and more attention has focused on emerging TBPs and ticks. Additionally, a wide variety of pathogenic and non-pathogenic bacteria have been identified. In this study, the bacterial community diversity of *Haemaphysalis* spp. in Shanghai was analyzed and compared based on the 16S rDNA high-throughput sequencing technique combined with nested PCR to survey TBPs in *Haemaphysalis* spp. collected from Shanghai, China. It is found that the 16S rDNA V3–V4 variable region sequence of bacteria can effectively detect the bacterial community composition and diversity of *H. flava* and *H. longicornis*. At the same time, sequencing data can also be used to evaluate the relative abundance of bacteria and the differences in flora structure between different groups.

In this study, two species of *Haemaphysalis* ticks: *H. longicornis* and *H. flava*, were collected. *H. flava* often lives in mixed forests and fields, and their hosts are mainly pigs, pig badgers, horses and sheep, while *H. longicornis* mainly live in secondary forests, mountains, or hilly marginal areas, and their hosts are mainly cattle, horses, sheep, goats, bears, hedgehogs, etc.) [22]. However, in the past, *H. longicornis* was the main tick species in Shanghai [21], but today, *H. flava*, which has spread all over Shanghai, is the main tick species. In light of the ongoing geographical expansion of ticks caused by climatic change, as well as their increased ability to harbor new pathogens, public health concerns have been raised for both humans and animals [21,30,31,32]. Many new tick-borne pathogens have been discovered in recent decades, indicating the serious public health threat that tick-borne diseases have imposed on China [30]. The main pathogens reported in *H. flava* are *Ehrlichia*, *Rickettsia japonica*, Crimean–Congo hemorrhagic fever virus (CCHFV), *Pseudomonas aeruginosa* and *Rickettsia raoultii* [9,30]. Additionally, *H. longicornis* carries pathogens such as SFTSV, *Anaplasma*, spotted fever group *Rickettsia* (SFGR), *Babesia*, etc. [33,34]. The number of species of *Haemaphysalis* ticks in Shanghai is small, but its potential harm to human and animal husbandry should not be ignored.

In this study, the diversity of bacterial communities of *H. flava* and *H. longicornis* was different in species and regions. The α diversity analysis of metagenomics also showed that the combinations of tick bacteria were different with different biological factors (such as different developmental stages, age and sex) and differed from the tissue and environmental conditions investigated in other studies [17,35,36]. However, some regional differences were detected in most of the metagenomics β diversity indexes of male and female ticks [17], and any regional and gender differences were also found in the relative abundance of different bacterial groups [35,36,37,38]. The core bacterial communities may be influenced by maternal inheritance, the vertebrate animal skin microflora, host blood or the environment on physical contact [39,40]. However, a more specific and long-term association between some blood-feeding arthropods and their bacterial associate is likely mediated by the immune system of the host rather than by their external environment [40,41,42]. Bacterial taxa such as *Bacillus*, *Clostridium*, *Methylobacterium*, *Mycobacterium*, *Pseudomonas*, *Sphingomonas* and *Staphylococcus*, some of which were found in high abundance in our samples, contain species that may be commonly associated with the environment or as part of the mammalian skin flora [3,40,41,42]. Therefore, more stringent washing procedures, such as washing with sodium hypochlorite, or limiting sampling to internal organs, such as the salivary glands and mid-gut, may be necessary to determine the internal flora of ticks in further studies [3].

The bacteria in all the arthropod species were dominated by the phylum Proteobacteria, with proportions ranging from 48% to 72%, and their major bacteria phyla that were shared among all the arthropod species included Firmicutes, Bacteroidetes and Actinobacteria [40]. Our results are generally similar to those obtained in previous studies, where arthropod vectors species were dominated by Proteobacteria, including Gammaproteobacteria, Betaproteobacteria, Alphaproteobacteria, and to a lesser extent, Firmicutes, commonly Bacilli and Actinobacteria [40,43,44,45,46]. The taxonomic profiles at the genus level confirmed that genera *Rickettsia*, *Anaplasma*, *Ehrlichia* and *Coxiella* existed in the pooled sample, which all predominated in the top six in relative abundance [30]. The mutual connection of symbiotic bacteria hosts exists widely in nature. In recent years, the relationship between symbiotic bacteria and the host has received more and more attention; especially, the mode and mechanism of interaction between symbiotic bacteria and the host have aroused widespread concern. A past study has shown that *Coxiella*-like bacteria are frequently detected in *Haemaphysalis* ticks, although not all species carry them, and *Coxiella*-like endosymbionts have also been reported in other tick species such as *H. shimoga*, *Rhipicephalus sanguineus*, *Amblyomma americanum*, and the soft tick, *Ornithodoros rostratus* [3,47,48,49,50]. The significance of *Coxiella* in tick physiology is still unclear. Ticks may depend on bacteria, such as *Coxiella*-like bacteria and *Coxiella*-like endosymbionts (CLE), which play important roles in the processes of food digestion, biosynthesis, growth and development, reproductive regulation and the immune defense of ticks [51]. Studies have confirmed that the CLE genome has complete coding genes of the key coenzyme factor (B vitamins) biosynthesis pathway, which provides a stable source of vitamins for host ticks [52,53]. Removing CLE will significantly reduce the fertility of host ticks [53,54]. The host tick provides CLE with a stable growth environment and nutrition, which makes CLE grow and propagate in ticks and spread to offspring ticks. However, not all individuals of the same tick species will harbor endosymbionts at a similar abundance; some individuals may not carry any *Coxiella*-like endosymbionts at all according to other studies [3,54].

*Coxiella*-like bacteria, *Legionella*, *Sphingomonas* and other strains were detected. *Coxiella*-like bacteria are common in all tick samples, and the content is very high, which indicates that it may be an endosymbiont [55]. So far, in addition to focusing on the study of microbial communities and specific symbionts in ticks, attention has also been paid to bacterial interactions in ticks. There was a negative correlation between symbiotic bacteria and the serous edge of pathogens and a positive correlation among endosymbionts, *Clostridium novyi* and *Corynebacterium cereus* [56]. There also is a repulsion between symbiotic *Rickettsia* and pathogenic *Rickettsia* [35,57]. A detailed understanding of the interaction among microflora in ticks is helpful in developing new strategies for pathogens and tick vector control [17].

After sequencing the DNA fragments amplified by PCR and the sequence comparison, three *Rickettsia* species (*Candidatus R. jingxinnensis*, *R. rickettsii* and *R. japonica*), one *Anaplasma* species (*A. bovis*), two *Coxiella* species (*Coxiella* spp. and *C.*-like endosymbiont) and two *Ehrlichia* species (*E. chaffeensis*, *E. ewingii*) were found in both *H. flava* and *H. longicornis*. In Jiangsu province, China, which is close to Shanghai, Chian, *R. japonica* (81.1%), novel *Rickettsia* spp. (5.1%), *A. bovis* (12%), *A. platys* (6.3%), novel *Ehrlichia* spp. (16.6), *C. burnetii* (10.9%), and a novel *Coxiella*-like endosymbiont (CLE) strain (61.1%), detected in *H. flava* [30], are higher than in Shanghai, China. Especially, *R. japonica* has still been prevalent in China and Japan in recent years and can cause Oriental spotted fever. Interestingly, *H. flava* had a high positive rate (81.1%) of *R. japonica* in Jiangsu province, China [31], compared to the 3.32% detected in this tick species in Shanghai, China, in this study.

At present, there are three known species of *Rickettsia japonica*, *Rickettsia rickettsii* and *Candidatus Rickettsia jingxinensis* in Shanghai, as well as new species of *Rickettsia* that have not been identified or cultivated successfully, which have certain public health risks. Japanese spotted fever (JSF) caused by *Rickettsia japonica* infection is an acute febrile eruptive disease with ticks as the vector. The pathogen can also be detected in *H. longicornis* collected in Shandong, and researchers in Fujian province, China, have also amplified highly similar genes of *Rickettsia japonica*. Japan and South Korea have reported confirmed cases of Japanese spotted fever, and there are reports of *Rickettsia japonica* infection in Hebei, Anhui provinces, China [58]. Rocky Mountain Spotted Fever (RMSF) is caused by *R. rickettsii* infection. It has been observed that *Candidatus R. jingxinensis* can infect humans, showing the clinical features of fever, erythema rash and eschar [59]. Previous studies have shown that the main vectors of *Rickettsia japonica* are *H. flava*, *H. formosensis*, *H. longicornis*, *H. cornigera*, *Ixodes ovatus*, *Rhipicephalus haemaphysaloides* and *Dermacentor taiwanensis*. The vectors of RMSF are *Amblyomma sculptum*, *Amblyomma aureolatum* and *Dermacento anderson*. *Candidatus R. jingxinensis* were also detected in *H. longicornis* in Yunnan, Liaoning, Hebei and Jiangsu provinces, China, and Korea [60,61]. In Shanghai, *Candidatus R. jingxinensis* was detected in not only *H. longicornis* but also *H. flava*. In this study, *Candidatus R. jingxinensis* was detected from two tick species of *Haemaphysalis* spp., especially *H. flava*, which has spread all over Shanghai, China, which indicated that *Candidatus R. jingxinensis* had the characteristics of wide distribution. The deficiency of this study is that all three *Rickettsia* species have been detected in new tick species, but the genotype cannot be further confirmed and isolated by culture.

The two predominant human pathogens within the *Ehrlichia* genus are *E. chaffeensis*, the etiologic agent of human monocytic ehrlichiosis (HME) distributed in the United States, Asia and Europe, and *E. ewingii*, the agent of human *E. ewingii* granulocytic ehrlichiosis [62,63,64]. In southern and northern China, clues of *E. chaffeensis* were found, which indicated that *E. chaffeensis* might exist in China [65,66]. *E. ewingii*, one of the causative agents of canine granulocytic ehrlichiosis, has been reported in dogs and humans in the USA [67]. However, previous investigations and studies in China have found no clues of *E. ewingii*.

*A. bovis* is mainly distributed in Asia and South America. Cattle and buffalo are considered the main hosts of *A. bovis*, but *A. bovis* has also been found in dogs in China. The intangible disease is a tick-borne disease that is mainly prevalent in tropical and subtropical areas. It is not only healthy for livestock. Additionally, it will also threaten human health. Qin [41] investigated in Jiaonan, Shandong Province, China, the infection rate of the blood of ticks was found to be 0.10% for *A. pagocytophilum*, 1.55% for *A. bovis* and 0.33% for *A. capra* [68].

This study has shown that high-throughput 16S rDNA sequencing analysis can detect multiple pathogens at the same time, which can indicate the main local tick-borne pathogens, but it also has the disadvantage of incomplete detection and is unable to match species. Combined with pathogen-specific detection methods, we can more comprehensively understand the status of local *Haemaphysalis* spp.-carrying pathogens, so we need to combine PCR and metagenomics analysis.

In this study, it is worth noting that *Serratia* was detected in the medical tick group. *Serratia* infection can activate the mosquito’s immune system, significantly improve the mosquito’s resistance to *Plasmodium berghei* infection and reduce the malaria parasite load in Anopheles. A recent research report showed that *Serratia* isolated in the laboratory could promote insect-borne virus infection in mosquitoes [69,70]. This bacterium secretes a protein called SmEnhancin, which can bind mucin on intestinal mucosa to promote the colonization and spread of the virus in mosquitoes [71]. However, the research on ticks is blank, so we need to further study the interaction between ticks, hosts and the bacterial communities in ticks and pathogens and further explain their interaction mechanism.

## 5. Conclusions

This study reports on the bacterial communities and the prevalence of some TBPs in *Haemaphysalis* spp. from Shanghai, China. The results provide insight into the potential roles of *Haemaphysalis* ticks in the epidemiology of pathogens of veterinary and public health significance. Further studies are needed to elucidate the implications of these findings on animals and humans in Shanghai, China. However, these findings are preliminary. Further studies are required to determine the interaction and roles of some of the bacterial communities in tick survival and vector competence.

## Figures and Tables

**Table 2:** Prevalence of *Rickettsia*, *Anaplasma*, *Ehrlichia* and *Coxiella* in *H. flava* and *H. longicornis*.

	*H. flava*	*H. longicornis*
*R. rickettsii*	2.37% (5/211)	20.00% (3/15)
*R. japonica*	3.32% (7/211)	20.00% (3/15)
*Candidatus R. jingxinensis*	16.59% (35/211)	20.00% (3/15)
*A. bovis*	1.42% (3/211)	26.67% (4/15)
*E. ewingii*	0.95% (2/211)	13.33% (2/15)
*E. chaffeensis*	1.90% (4/211)	6.67% (1/15)
*Coxiella* spp.	1.90% (4/211)	6.67% (1/15)
*C.*-like endosymbiont	2.37% (5/211)	20.00% (3/15)

## Data Availability

The DNA sequencing data has been uploaded to GeneBank.
